# A Hand-Book of Climatic Treatment, Including Balneology

**Published:** 1906-06

**Authors:** 


**A Hand-book of Climatic Treatment, including Balneology.** By WILLIAM R. HUGGARD, M.A., M.D. Pp. xvi., 536. London: Macmillan & Co. 1906.

So many books on climatic treatment have followed each other rapidly for some years past that it is a bold and ambitious undertaking to endeavour to surpass them, but the writer of this one, His Britannic Majesty's Consul at Davos, has had exceptional opportunities of working at the subject, and has collected materials steadily during many years. He has accordingly produced, as he might be expected to do, a standard work founded greatly on personal experience; his chief aim has been to place the therapeutics of climate on a secure foundation, and he founds his classification of climates on "*the demand made by the climate for the production of heat.* This corresponds with the demand for tissue change. Now the tonic or relaxing character of a climate turns chiefly on the ability of the organism to adapt itself to the requirements. Other things being equal, that climate is most tonic which demands the greatest amount of tissue change that a given organism can permanently yield." This quotation gives the keynote of the book, for temperature is the only meteorological factor that retains its place as an element of classification for all climates. Davos and the other Alpine resorts, and the high-lying resorts of the Rocky Mountains represent the climates where the heat demand (or demand for tissue change) is excessively large—the great high plateau of South Africa belongs essentially to this same group. One feature common to these regions is that the climate is a strong inducement to an open-air life, but the author admits that "no climate has a monopoly of good effect, and that for every climate there are unsuitable cases," accordingly "the patient may do well in any fairly good climate when he follows a suitable mode of life." These quotations show that the author's ideas are not limited by the interests of his own health resort, that he takes a fair, common-sense view of the questions with which he deals, and that his book may be taken as a trusty guide by all those who seek information on climatic and balneological questions.

